# Hepatic arterial infusion chemotherapy combined with PD-1 inhibitors and tyrosine kinase inhibitors for unresectable hepatocellular carcinoma: A tertiary medical center experience

**DOI:** 10.3389/fonc.2022.1004652

**Published:** 2022-09-23

**Authors:** Laihui Luo, Yongqiang Xiao, Guoqing Zhu, Aihong Huang, Shengjiang Song, Tao Wang, Xian Ge, Jin Xie, Wei Deng, Zhigao Hu, Wu Wen, Haoran Mei, Renhua Wan, Renfeng Shan

**Affiliations:** ^1^ Department of General Surgery, The First Affiliated Hospital of Nanchang University, Nanchang, China; ^2^ Department of Infectious Disease, The First Affiliated Hospital of Nanchang University, Nanchang, China; ^3^ Department of Day Surgery Ward, The First Affiliated Hospital of Nanchang University, Nanchang, China; ^4^ Department of Pathology, The First Affiliated Hospital of Nanchang University, Nanchang, China

**Keywords:** unresectable hepatocellular carcinoma, hepatic arterial infusion chemotherapy, programmed cell death protein-1, tyrosine kinase inhibitors, conversion therapy

## Abstract

**Background:**

Unresectable hepatocellular carcinoma (u-HCC) still accounts for the majority of newly diagnosed HCC which with poor prognosis. In the era of systemic therapy, combination therapy with programmed cell death protein-1 (PD-1) inhibitors and tyrosine kinase inhibitors (TKIs) has become mainstream. Hepatic arterial infusion chemotherapy (HAIC) as a local treatment has also shown a strong anti-tumor effect. This study aimed to investigate the efficacy and safety of HAIC, PD-1 inhibitors plus TKIs for u-HCC.

**Methods:**

This retrospective study included patients with initially u-HCC between October 2020 to April 2022 who had received at least one cycle of therapy with HAIC, PD-1 inhibitors plus TKIs. The primary outcome included overall response rate (ORR), the disease control rate (DCR), surgical conversion rate, progression-free survival (PFS) and treatment-related adverse events.

**Results:**

A total of 145 patients were included in the study. The median treatment cycle of HAIC and PD-1 inhibitors were 3 and 4, respectively. According to the modified RECIST criteria, the best ORR was 57.2% (83/145), 9 had achieved complete response (CR), DCR was 89.7% (130/145). Median time to achieve CR or PR was 65 days. Surgical conversion rate was 18.6% (27/145), seven patients (7/27,25.9%) achieved pathological complete response (pCR). The median follow-up was 12.5 months (4.5-20 months), and the median PFS was 9.7 months. Subgroup analysis showed that Child-pugh A patients had higher DCR (92.2% vs 79.3%, *p*=0.041) than Child-pugh B patients, as well as increased successful conversion rate (22.4% vs 3.4%, *p*=0.019). Patients without vascular invasion and extrahepatic metastases showed higher PR (63.4% vs 43.3%, *p*<0.05) and ORR (73.2% vs 50.0%, *p*<0.05) than those with vascular invasion. The ORR (73.2% vs 45.5%, *p*<0.05) and DCR (95.1% vs 78.8%, *p*<0.05) were also significantly better than those of patients with extrahepatic metastases. HAIC regimen was not related to efficacy (All *p*>0.05). The incidence rate of grade 3/4 treatment-related AEs was 17.7% without fatal events.

**Conclusion:**

The triple combination therapy of HAIC and PD-1 inhibitors plus TKIs for patients with initially unresectable HCC exhibited satisfactory efficacy with tolerable toxicity.

## Introduction

Hepatocellular carcinoma (HCC) is still the most common kinds of malignant tumors worldwide, although the morbidity is decreasing stably in China (1). The prognosis of HCC remains poor, and approximately 830000 newly deaths every year (2). Although progress has been made in early screening for HCC, the majority have lost the chance of cure at the time of diagnosis. These so called “unresectable HCC” (u-HCC) have worse prognosis with the median overall survival (OS) ranging from 1 to 2 year (3).

Systemic therapy is the preferred option for u-HCC patients with the advent of sorafenib, but the objective response rate (ORR) remains far from satisfactory. With the publications of REFLECT, RESORCE, CELESTIAL and REACH-2 study, more novel tyrosine kinase inhibitors (TKIs) have been the alternative modality of sorafenib, such as lenvatinib, apatinib, cabozantinib, and ramucirumab, but the outcomes remain poor with the ORR of 4% to 18.8% (4). Trial of CheckMate040 has ushered the era of immunotherapy for HCC in the recent years, but phase III CheckMate459 trials of PD-1 inhibitors monotherapy for HCC have all failed to meet the primary endpoints (5). Dual combination regimen, such as lenvatinib plus pembrolizumab, camrelizumab plus apatinib, and sintilimab plus anlotinib, have yielded promising clinical efficiency and safety, but the prognosis for those u-HCC patients is still unsatisfactory with the median OS of 20.1 to 20.4 months (6–8).

IMbrave150 trial has not only opened the era of molecular target and immunotherapy, but also shed light on the triple combination of local regional therapy, TKIs, and immunotherapy. In the trial of IMbrave150, about 40% patients received previous transarterial chemoembolization (TACE) before enrollment (9). In the recent years, hepatic arterial infusion chemotherapy (HAIC) has been identified to be alternative strategy of TACE in the management of advanced HCC, which has the advantage over TACE for those with extrahepatic metastasis or macrovascular invasion (10, 11). Combination of HAIC and sorafenib has exhibited significant survival benefit compared with sorafenib or HAIC alone, but the 2-year survival rate remains low (12, 13).

In addition, “conversion therapy” has been well concerned in the field of HCC, which needs a more aggressive strategy. In the past two years, triple therapy of TACE/HAIC, TKIs, and immune checkpoint inhibitors (ICIs) have been tried with encouraging results, but most of the studies were retrospective with small sample size. In this study, we aimed to evaluate the clinical efficacy and safety of the triple regimen of HAIC, PD-1 inhibitors and TKIs for u-HCC patients in a retrospective study of single-center.

## Patients and methods

### Patients selection

All consecutive patients in our hospital diagnosed as u-HCC and received triple therapy of HAIC, PD-1 inhibitors and TKIs from October 2020 to April 2022 were enrolled in this study. The exclusion criteria were as followed: 1) age <18 years old, 2) recurrent HCC, 3) receiving other antitumor treatment, 4) postoperative adjuvant HAIC, 5) terminated treatment, and 6) without treatment evaluation. Of note, the definition of u-HCC in this study was oncologically or biologically unresectable: technically resectable, but resection does not result in a better outcome than non-surgical treatment.

This study was approved by the Ethics Committee of The First Affiliated Hospital of Nanchang University, Nanchang, China. No (2022). CDYFYYLK (06–009). Considering that patients medical records were analyzed retrospectively and no patient-identifiable information was utilized, the ethics committee waived the need for individual consent.

### HAIC procedure

The procedure of HAIC was similar as previous report and the regimens in this study included FOLFOX (HAIC with oxaliplatin, 5-fluorouracil, and leucovorin) and RALOX (HAIC with raltitrexed plus oxaliplatin). Briefly, the femoral artery was punctured by the Seldinger technique after local anesthesia, then the blood supply of the tumor was determined using the digital subtraction angiography, and at last a 2.7-F microcatheter was maintained at the tumor-feeding arteries for HAIC. HAIC was carried out in the ward within two days, during which the microcatheter was connected externally to an artery infusion pump. Notably, the dose of drugs would be adjusted according to the Child–Pugh grade and tolerance to chemotherapy.

### Tyrosine kinase inhibitors and PD-1 inhibitors

Considering the accessibility of drugs, TKIs in this study were sorafenib, apatinib and lenvatinib, and PD-1 inhibitors were camrelizumab, stintilimab and tislelizumab. The dose of these agents was administrated according to the guidelines, which would also be adjusted according to the performance status, liver function, and treatment tolerance.

### Data collection

Collecting clinical data of patients during each hospital admission. Baseline clinical characteristics including: age, gender, Eastern Cooperative Oncology Group Performance Status (ECOG PS) score, positive or negative of hepatitis B surface antigen, liver cirrhosis, Child-Pugh classification, ALBI* grade, total bilirubin, albumin,α-fetoprotein (AFP) level, Barcelona Clinic Liver Cancer (BCLC) stage, Chinese Liver Cancer (CNLC) stage, American joint Committee on cancer (AJCC) stage, size of largest nodule, tumor number, tumor distribution, absence or presence of macroscopic vascular invasion (portal vein tumor thrombus or hepatic vein tumor thrombus), absence or presence of extrahepatic metastasis. At the same time, the imaging examination results of each patient were collected to evaluate the efficacy response. The patient’s follow-up treatment was also collected.

* ALBI: albumin-bilirubin; Calculated using the following equation: linear predictor = (log_10_ bilirubin μmol/L×0.66) + (albumin g/L×−0.085). The continuous linear predictor was further categorized into three different grades for prognostic stratification purposes: grade 1 (less than −2.60), grade 2 (between −2.60 and −1.39) and grade 3 (above −1.39) (14).

### Follow-up

The triple therapy was terminated if complete response (CR) was achieved, the patient received surgery, the disease progressed or the patient experienced intolerable toxicity. Blood examination including blood cell analysis, bio-chemistry, and AFP were performed before and after each cycle of treatment. Abdominal contrast-enhanced CT scan or MRI and chest computed tomography (CT) every cycle (4–6 weeks) after initial treatment. Patients were followed up every 3 months until death or censored.

### Outcomes

The primary end points of this study were safety and PFS. The complete response (CR), objective response rate (ORR), disease control rate (DCR) and successful conversion rate were also recorded.

The tumor response was assessed by two independent experienced radiologists according to modified Response Evaluation Criteria in Solid Tumors (mRECIST) criteria and RECIST 1.1 criteria based on the abdominal enhanced CT or MRI and chest CT, as well as AFP levels. CR was defined as the disappearance of all lesions or no enhancement by enhanced CT/MRI for at least 4 weeks and normal AFP levels. If there were discrepancies in the assessments between the two independent radiologists, another radiologist was asked to evaluate the response to determine the tumor response rate.

PFS was defined as the time from initial treatment to disease progression or death from any reason. Disease progression included intrahepatic tumor and/or extrahepatic tumor progression.

Safety was assessed among all the patients treated, and all the treatment-related adverse events (AEs) were determined by the National Cancer Institute Common Terminology Criteria for Adverse Events (version 5.0), but the immune-related AEs (irAEs) were diagnosed, managed and followed-up according to the European Society for Medical Oncology Clinical Practice Guidelines.

### Statistical analyses

Continuous variables including age, AFP level, ALBI grade, total bilirubin, albumin, tumor size, and tumor number were categorized as previously reported, all the variables in this study were presented as n (%). Survival analysis was calculated using the Kaplan-Meier method. Subgroup analysis was conducted stratified by different Child-pugh classification, BCLC stage and regimen. All the statistical tests were two-tailed, and *p*<0.05 was considered to be statistically significant. Statistical tests were conducted using RStudio, including the **Table 1**, survminer, rms, and survival packages.

**Table 1:** Baseline clinical characteristics.

Characteristics		No. (%)
Age, years		
	≤50	61 (42.1)
	>50	84 (57.9)
Gender		
	Male	121 (83.4)
	Female	24 (16.6)
ECOG		
	0	138 (95.2)
	1	7 (4.8)
Hepatitis B virus infection		
	Positive	133 (91.7)
	Negative	12 (8.3)
Liver cirrhosis		
	Absent	30 (20.7)
	Present	115 (79.3)
Child-Pugh classification		
	A	116 (80.0)
	B	29 (20.0)
ALBI grade		
	1	36 (24.8)
	2	104 (71.7)
	3	5 (3.4)
Total bilirubin (μmol/L)		
	≦̸20	88 (60.7)
	>20	57 (39.3)
Albumin (g/L)		
	<40	114 (78.6)
	≧̸40	31 (21.4)
AFP (ng/ml)		
	≦̸400	63 (43.4)
	>400	82 (56.6)
BCLC stage		
	B	41 (28.3)
	C	104 (71.7)
CNLC stage		
	IIa	5 (3.4)
	IIb	36 (24.8)
	IIIa	71 (50.0)
	IIIb	33 (22.8)
AJCC stage		
	II	8 (5.5)
	IIIA	33 (22.8)
	IIIB	65 (44.8)
	IVA	22 (15.2)
	IVB	17 (11.7)
Size of largest nodule (cm)		
	<10	72 (49.7)
	≥10	73 (50.3)
Tumor number		
	Solitary	37 (25.5)
	Multiple	108 (74.5)
Tumor distribution		
	Uni-lobar	71 (49.0)
	Bi-lobar	74 (51.0)
Macrovascular invasion		
	Absent	70 (48.3)
	Present	75 (51.7)
	PVTT	66
	HVTT	7
	PVTT+HVTT	2
Extrahepatic metastasis		
	Absent	112 (77.2)
	Present	33 (22.8)

ECOG; Eastern Cooperative Oncology Group. ALBI, albumin-bilirubin; Calculated using the following equation: linear predictor = (log_10_ bilirubin μmol/L×0.66) + (albumin g/L×−0.085). The continuous linear predictor was further categorized into three different grades for prognostic stratification purposes: grade 1 (less than −2.60), grade 2 (between −2.60 and −1.39) and grade 3 (above −1.39);AFP, α-fetoprotein; BCLC, Barcelona Clinic Liver Cancer; CNLC, China Liver Cancer; AJCC, American Joint Committee on Cancer; PVTT, Portal vein tumor thrombus; HVTT, Hepatic vein tumor thrombus.

## Results

### Patient clinic characteristics

**Figure 1:**
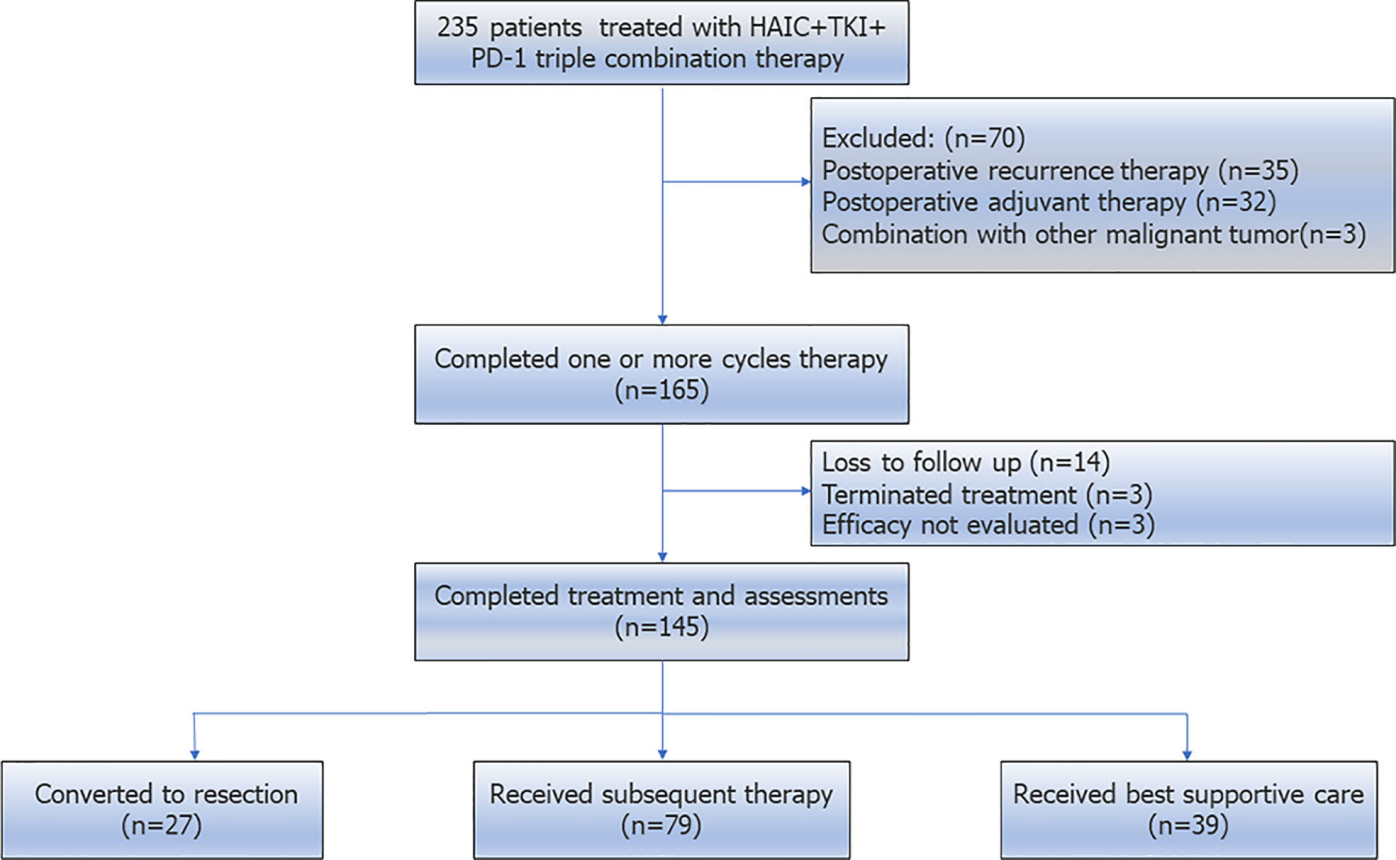
Patient selection flow. Initially, 235 patients receiving HAIC+TKIs+PD-1 were identified. And at last, 145 patients were eligible for further analysis.

Initially, 235 patients receiving HAIC+PD-1+TKIs were identified. 90 patients were excluded as followed: 1) receiving postoperative recurrence therapy (n=35), 2) receiving postoperative adjuvant therapy (n=32), 3) combined with other types of malignant tumor (n=3), 4) lost to follow up (n=14),5) terminated treatment (n=3) and 6) efficacy not evaluated (n=3). And at last, 145 patients were eligible for further analysis (**Figure 1**).

The baseline characteristics of the included patients were depicted in **Table 1**. Of note, 29 (20%) patients were present with Child-Pugh B, 109 (75.1%) were with ALBI grade 2 and 3, 75 (51.7%) were with macrovascular invasion, and 33 (22.8%) were with extrahepatic metastasis.

**Table 2** exhibited the regimens of the triple combination therapy, including the HAIC regimen, PD-1 inhibitors scheme and TKIs prescription. Briefly, the median cycle teratment of HAIC and PD-1 inhibitors were 3 and 4, respectively. Specially, TKIs and PD-1 inhibitors regimens were diverse,lenvatinib+camrelizumab (n=91), lenvatinib+stintilimab (n=16), lenvatinib+tislelizumab (n=6), sorafenib+camrelizumab (n=19), sorafenib+stintilimab (n=4), and apatinib+camrelizumab (n=9), respectively.

**Table 2:** Regimens of the triple combination therapy.

Treatment		No. (%)
HAIC regimen	FOLFOX	113 (77.9)
	RALOX	32 (22.1)
TKI+PD-1 regimen	Lenvatinib+Camrelizumab	91 (62.8)
	Lenvatinib+ Stintilimab	16 (11.0)
	Lenvatinib+ Tislelizumab	6 (4.1)
	Sorafenib+ Camrelizumab	19 (13.1)
	Sorafenib+ Stintilimab	4 (2.8)
	Apatinib+ Camrelizumab	9 (6.2)
HAIC treatment cycle		
	Median (range)	3 (1-6)
PD-1 treatment cycle		
	Median (range)	4 (1-10)

HAIC, hepatic arterial infusion chemotherapy; FOLFOX, HAIC with oxaliplatin, 5-fluorouracil, and leucovorin; RALOX, HAIC with raltitrexed plus oxaliplatin; TKIs, tyrosine kinase inhibitors; PD-1, programmed cell death protein-1.

### Outcomes

**Figure 2:**
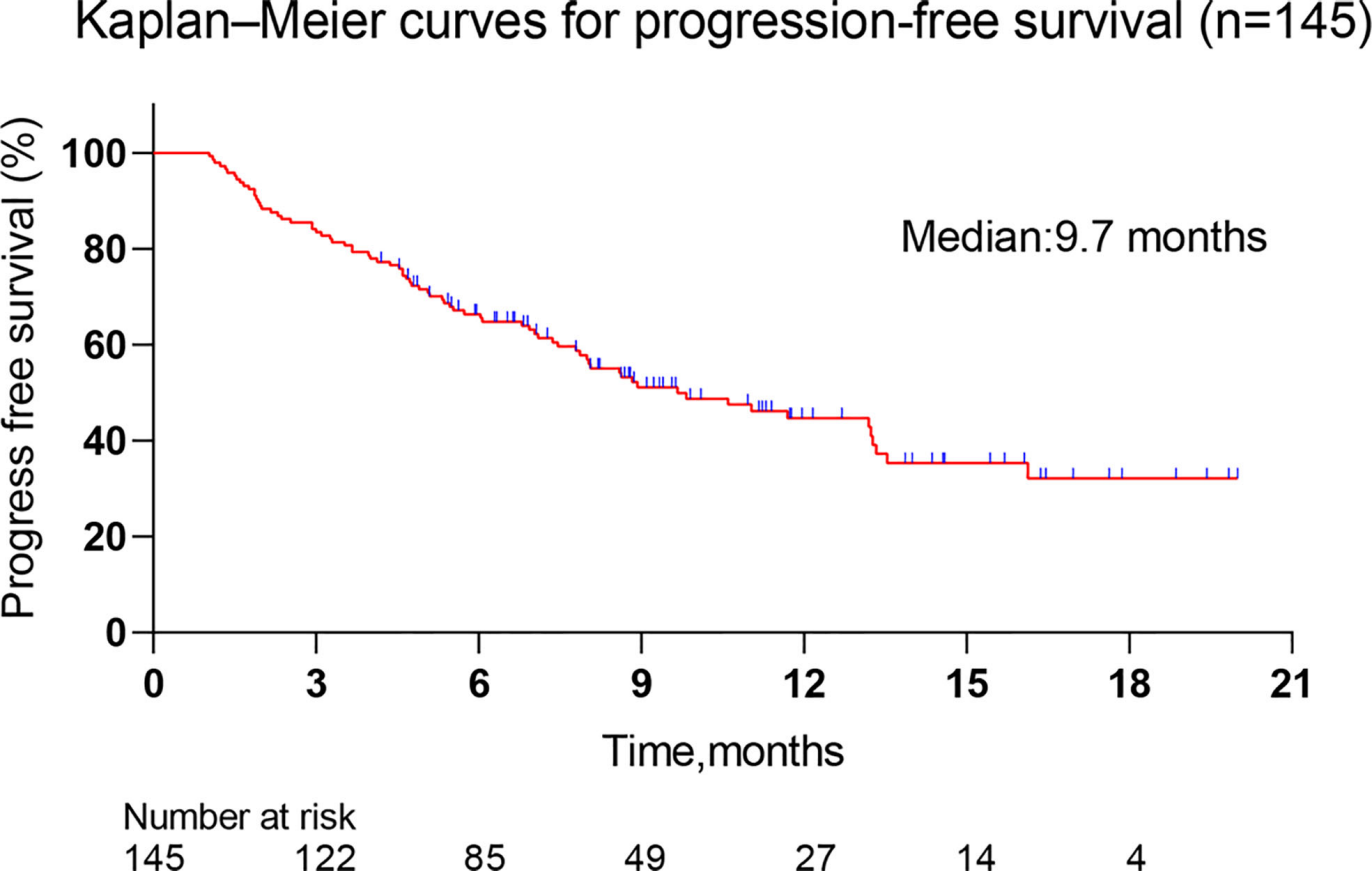
Kaplan–Meier curves for progression-free survival. The median follow-up was 12.5 months (4.5-20 months), and the median PFS was 9.7 months (1-16.1 months).

The median follow-up was 12.5 months (4.5-20 months), and the median PFS was 9.7 months (1-16.1 months, **Figure 2**). The corresponding PFS rates at 6-months, 9-months, 12-months and 15-months were 66.9%, 55.2%, 51.7%, and 48.3%, respectively.

**Figure 3:**
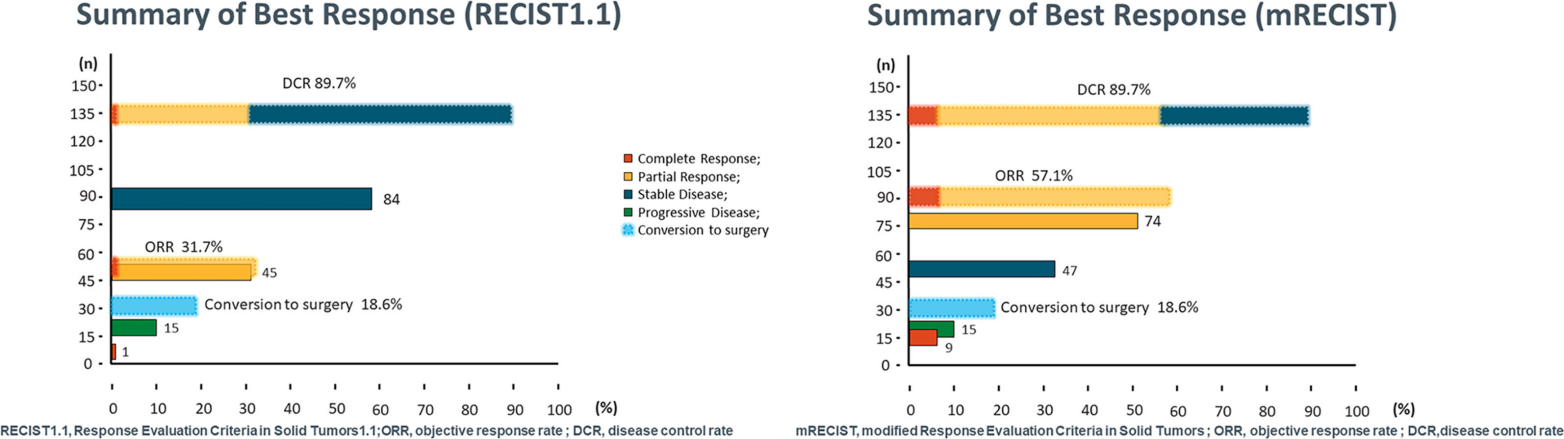
Summarized the results of the best response according RECIST1.1 and mRECIST criteria. RECIST1.1, Response Evaluation Criteria in Solid Tumors1.1; mRECIST, modified Response Evaluation Criteria in Solid Tumors.

**Figure 4:**
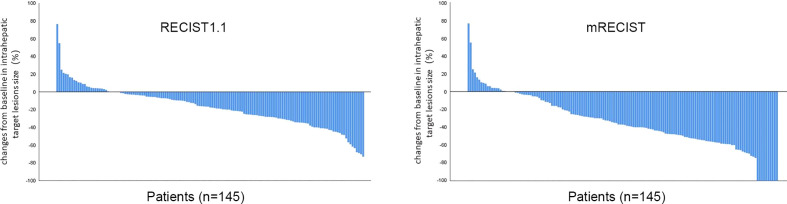
Best percentage changes from baseline in size of the intrahepatic target lesions of patients. RECIST1.1, Response Evaluation Criteria in Solid Tumors1.1; mRECIST, modified Response Evaluation Criteria in Solid Tumors.

**Figure 3** summarized the results of the best response (mRECIST and RECIST 1.1). For consistency, tumor response was only depicted using mRECIST in the following section. During the follow-up period, 9 (6.2%) patients achieved CR, 74 (51%) achieved PR, 47 (32.5%) achieved SD, and 15 (10.3%) achieved PD. The ORR reached 57.2%, as well as increased DCR of 89.7%. The median time to achieve CR or PR was 65 days (21-175 days). A waterfall plot showed the change in the intrahepatic target lesion size of patients (**Figure 4**).

Twenty-seven patients received surgery, and the successful conversion rate was 18.6% (27/145) with the median duration of triple therapy was 91 days. All 27 patients underwent open hepatectomy and recovered well after surgery. Two patients developed serious complications, including postoperative liver failure, massive pleural effusion, and dyspnea, but were recovered after symptomatic treatment. All (100%) patients achieved R0 resection, and 7 (25.9%) were confirmed to achieve pathological CR (pCR). Until June 2022, 9 (33.3%) patients had tumor recurrence or metastasis.

### Subgroup analysis

According to Child-Pugh classification, 116 (80.0%) patients were graded A and 29 (20.0%) were B, respectively. Subgroup analysis showed that the best response was in favor of patients with Child-Pugh A in terms of PD, DCR, and successful conversion rate compared with those with Child-Pugh B (all *p*<0.05, **Table 3A**).

**Table 3A:** Subgroup analysis according to liver function (Child-pugh A vs Child-pugh B).

	Child-pugh A (n = 116)	Child-pugh B (n = 29)	*p* value
	mRECIST		
**Best response (n, %)**			
CR	9 (7.8)	0 (0)	0.205
PR	60 (51.7)	13 (44.8)	0.506
SD	38 (32.7)	10 (34.5)	0.860
PD	9 (7.8)	6 (20.7)	0.041
ORR (CR+PR)	69(59.5)	13 (44.8)	0.154
DCR (CR+PR+SD)	107 (92.2)	23 (79.3)	0.041
**Conversion rate (n, %)**	26 (22.4)	1 (3.4)	0.019

mRECIST, modified Response Evaluation Criteria in Solid Tumors; CR, complete response; PR, partial response; SD, stable disease; PD, progressive disease; ORR, objective response rate; DCR, disease control rate.

In this study, 60 patients with macrovascular invasion (no extrahepatic metastasis), 33 patients had extrahepatic metastasis. Results showed that the best PR (63.4% vs 43.3%, *p*<0.05) and ORR (73.2% vs 50.0, *p*<0.05) in the subgroup of patients with macrovascular invasion was significantly lower than that without macrovascular invasion. The ORR (73.2% vs 45.5%, *p*<0.05) and DCR (95.1% vs 78.8%, *p*<0.05) of patients without extrahepatic metastases were significantly higher than those with extrahepatic metastases (**Table 3B**).

**Table 3B:** Subgroup analysis according to macrovascular invasion and extrahepatic metastasis (Group A vs Group B; Group A vs Group C).

	Group A(n = 41)	Group B(n = 60)	*p* value	Group A(n = 41)	Group C(n = 33)	*p* value
	mRECIST			mRECIST		
**Best response (n, %)**						
CR	4 (9.8)	4 (6.7)	0.712	4 (9.8)	1 (3.0)	0.373
PR	26 (63.4)	26 (43.3)	0.047	26 (63.4)	14 (42.5)	0.072
SD	9 (21.9)	25 (41.7)	0.039	9 (21.9)	11 (33.3)	0.273
PD	2 (4.9)	5 (8.3)	0.698	2 (4.9)	7 (21.2)	0.033
ORR (CR+PR)	30 (73.2)	30 (50.0)	0.020	30 (73.2)	15 (45.5)	0.015
DCR (CR+PR+SD)	39 (95.1)	55 (91.7)	0.698	39 (95.1)	26 (78.8)	0.033
**Conversion rate (n, %)**	11 (26.8)	9 (15.0)	0.143	11 (26.8)	5 (15.2)	0.280

Group A: No macrovascular invasion and extrahepatic metastasis; Group B: Macrovascular invasion (no extrahepatic metastasis); Group C: Extrahepatic metastasis.

mRECIST, modified Response Evaluation Criteria in Solid Tumors; CR, complete response;PR, partial response; SD, stable disease; PD, progressive disease; ORR, objective response rate;DCR, disease control rate.

According to the regimen of HAIC, 113 (77.9%) patients received FOLFOX regimen and 32 (22.1%) received RALOX regimen, respectively. No differences were observed between the two subgroups in terms of all the best response (all *p*>0.05, **Table 3C**).

**Table 3C:** Subgroup analysis according to HAIC regimen (FOLFOX vs RALOX).

	FOLFOX (n = 113)	RALOX(n = 32)	*p* value
	mRECIST		
**Best response (n, %)**			
CR	5 (4.4)	4 (12.5)	0.109
PR	59(52.2)	14 (43.8)	0.398
SD	36 (31.9)	12 (37.5)	0.549
PD	13 (11.5)	2 (6.2)	0.523
ORR (CR+PR)	64 (56.6)	18 (56.3)	0.670
DCR (CR+PR+SD)	100 (88.5)	30 (93.8)	0.523
**Conversion rate (n, %)**	22 (19.5)	5 (15.6)	0.622

FOLFOX: HAIC with oxaliplatin, 5-fluorouracil, and leucovorin; RALOX: HAIC with raltitrexed plus oxaliplatin; mRECIST, modified Response Evaluation Criteria in Solid Tumors; CR, complete response; PR, partial response; SD, stable disease; PD, progressive disease; ORR, objective response rate; DCR, disease control rate.

### Adverse events

**Figure 5:**
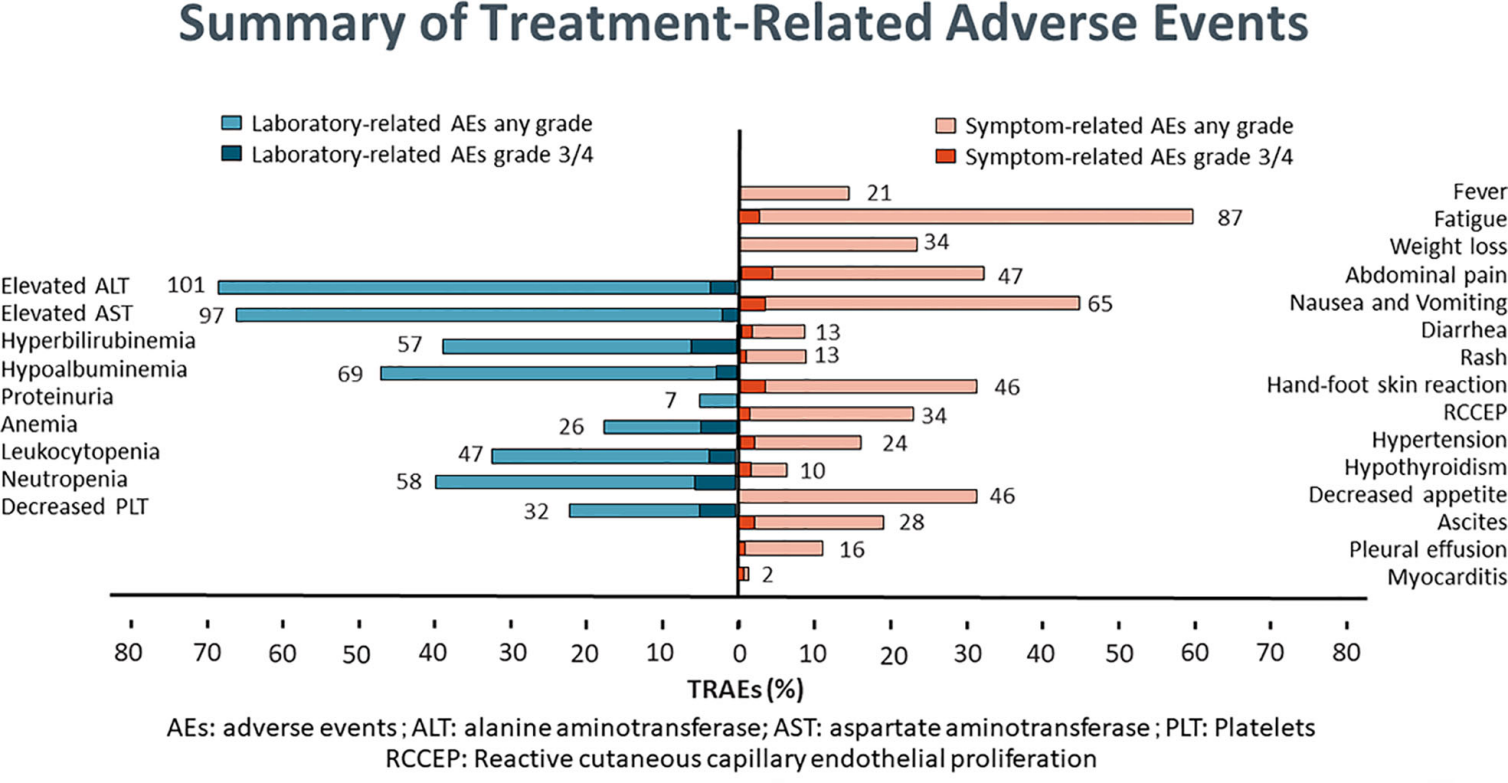
Summary of treatment-related adverse events.

The majority of patients experienced treatment-related AEs (**Figure 5**), but most of the AEs were mild or curative after treatment. The top three most common treatment-related AEs were elevated ALT, elevated AST, and fatigue, respectively. The incidence of grade 3/4 AEs were 17.7%, but none of the fatal AEs was reported. The top three most common grade 3/4 AEs were hyperbilirubinemia, myelosuppression, and abdominal pain, respectively.

### Subsequent therapy

A total of 79 patients continued to receive follow-up treatment (**Table 4**). Systemic therapy was still the first choice for these patients, the vast majority of patients continued to combine targeted and immunotherapy, and some patients added local therapy (TACE, HAIC). For metastases, microwave ablation and radiation therapy were also options.

**Table 4:** Subsequent therapy.

Subsequent therapy	N=79
TKI	8 (10.1)
PD-1	5 (6.3)
HAIC	5 (6.3)
TKI+PD-1	30 (37.9)
MWA+TACE	1 (1.3)
RFA+ Radiotherapy	1 (1.3)
TACE+ TKI+PD-1	9 (11.4)
HAIC+ TKI+PD-1	8 (10.1)
MWA+ TKI+PD-1	1 (1.3)
TKI+PD-1+ Radiotherapy	3 (3.8)
HAIC+TACE+ TKI+PD-1	4 (5.0)
TACE+ TKI+PD-1+Radiotherapy	1 (1.3)
TACE+ TKI+PD-1+Radiotherapy	1 (1.3)
MWA+ TKI+PD-1+ Radiotherapy	1 (1.3)
HAIC+TACE+ TKI+PD-1+Radiotherapy	1 (1.3)

TKIs, tyrosine kinase inhibitors; PD-1, programmed cell death protein-1; HAIC, hepatic arterial infusion chemotherapy; MWA, microwave ablation; TACE, transcatheter arterial chemoembolization. RFA, radiofrequency ablation.

## Discussion

In this study, we reported 145 patients with initially unresectable HCC who received HAIC plus PD-1 inhibitors and TKIs with CR of 6.2%, ORR of 57.2%, DCR of 89.7%, and successful conversion rate of 18.6%. The median follow-up was 12.5 months, and the median PFS was 9.7 months. In addition, the triple combination therapy regimen has controllable toxic and side effects.

In the era of lack of systemic therapy, TACE is the main means of conversion therapy for u-HCC with the ORR of 12.0% 18.1% (15). With the publication of IMbrave150 trial, the treatment combination of TKIs and ICIs has become mainstream, and the ORR has also reached 28.1%-38.6% (9). In addition, IMbrave150 also gave birth of an aggressive treatment for u-HCC, triple modality of arterial directed therapy (ADT), ICIs, and TKIs. Zheng, et al (16) firstly reported the combination treatment of TACE+sorafenib+ICIs for 29 u-HCC patients, and results showed that the triple therapy exhibited significant advantage over TACE+sorafenib in terms of DCR (81.82% vs. 55.17%, *p* = 0.046). This finding was verified by the subsequent studies and was also confirmed in the latest systematic review.

Recently, HAIC-based combination therapy has received increasing attention due to encouraging tumor response rates and patient survival rates. **Table 5** depicted all the published reports of combination of HAIC and TKIs plus ICIs. The published studies were almost come from small size sample of 25 to 84, and the results varied greatly from each study with the CR ranging from 0% to 48%, ORR from 40% to 96%, and DCR from 77.6% to 100%, respectively (17–21). This divergence might be contributed to the difference in study population (macrovascular invasion or not, extrahepatic metastasis or not) and therapy regimen (sorafenib, apatinib or lenvatinib, toripalimab, sintilimab, pembrolizumab or camrelizumab). In the present study of 145 patients in a single center, the corresponding CR, ORR, and DCR were 6.1%, 57.2% and 89.7%, respectively. And the median PFS was 9.7 months in the whole cohort, but with 18.6% achieved successful conversation. These findings were coincident with previous studies, but limitations were: 1) the heterogneity of study population, 51.7% were present with macrovascular invasion and 22.8% were with extrahepatic metastasis; 2) the divergence of liver function, the ALBI ranged from grade 1 to 3 regard less of 80% grading Child-Pugh A.

**Table 5:** Published literature for HCC patients received HAIC+TKIs+PD-1 treatment.

Study (Year), Design	Treatment	Patients	BCLC stageB/C	HAIC	TKIs	PD-1/PD-L1	CR	ORR	DCR	Conversion rate	MedianOS (months)	MedianPFS(months)	AE rate(Grade≥3)
1 (2021), Retrospective	HAIC+TKIs+PD-1	25	0/25	FOLFOX	Apatinib Lenvatinib Sorafenib	Camrelizumab Sintilimab	48%	96%	100%	56%	Not reached (median follow-up 12.53)	not reached	28%
2 (2021), Retrospective	HAIC+Lenvatinib+ Toripalimab Lenvatinib	71 86	0/71 0/86	FOLFOX	Lenvatinib	Toripalimab	14.1% vs 0%	67.6% vs 14.3%	90.1% vs 72.1%	/	Not reached vs 11	11.1 vs 5.1	Combination therapy group higher
3 (2021), Retrospective	HAIC+PD1+Lenvatinib Lenvatinib+PD-1	45 25	5/40 3/22	FOLFOX	Lenvatinib	Nivolumab Keytruda Toripalimab Sintilimab	0% vs 0%	40.0% vs 16.0%	77.6% vs 44.0%	/	15.9 vs 8.6	8.8 vs 5.4	22.2% vs 36.0%
4 (2021), Retrospective	HAIC+TKIs+PD-1	27	0/27	FOLFOX	Lenvatinib Regorafenib Sorafenib Apatinib	Camrelizumab Sintilimab Toripalimab Nivolumab	22.2%	63.0%	92.6%	/	Not reached (median follow-up 12.9)	12.9	55.6% (All grade 3)
5(2021), Retrospective	HAIC+ Pembrolizumab+ Lenvatinib Lenvatinib+Pembrolizumab	84 86	22/62 21/65	FOLFOX	Lenvatinib	Pembrolizumab	15.5% vs 9.3%	59.5% vs 41.9%	89.3% vs 86.1%	/	17.7 vs 12.6	10.9 vs 6.8	4.8% vs 2.3%
6 Ours, Retrospective	HAIC+PD-1+TKIs	145	41/104	FOLFOX RALOX	Sorafenib Lenvatinib Apatinib	Camrelizumab Stintilimab Tislelizumab	6.1%	57.2%	89.7%	18.6%	not reached (median follow-up 12.5)	9.7	17.7%

1、Surgical Conversion for Initially Unresectable Locally Advanced Hepatocellular Carcinoma Using a Triple Combination of Angiogenesis Inhibitors, Anti-PD-1 Antibodies, and Hepatic Arterial Infusion Chemotherapy:A Retrospective Study.

2、Lenvatinib, toripalimab, plus hepatic arterial infusion chemotherapy versus Lenvatinib alone for advanced hepatocellular carcinoma.

3、Hepatic Arterial Infusion Chemotherapy Combined With PD-1 Inhibitors Plus Lenvatinib Versus PD-1 Inhibitors Plus Lenvatinib for Advanced Hepatocellular Carcinoma.

4、Real-world study of hepatic artery infusion chemotherapy combined with anti-PD-1 immunotherapy and tyrosine kinase inhibitors for advanced hepatocellular carcinoma.

5、Pembrolizumab plus Lenvatinib with or without hepatic arterial infusion chemotherapy in selected populations of patients with treatment-naive unresectable hepatocellular carcinoma exhibiting PD-L1 staining: a multicenter retrospective study.

TKIs, tyrosine kinase inhibitors; HAIC, hepatic arterial infusion chemotherapy; PD-1, programmed cell death protein-1; PD-L1, programmed cell death ligand-1FOLFOX Regiment, oxaliplatin+leucovorin+5-fluorouracil; RALOX Regiment, raltitrexed + oxaliplatin; BCLC, Barcelona Clınic Liver Cancer; CR, complete response; DCR, disease control rate; ORR, objective response rate.

The underlying mechanism of the synergistic antitumor effect of the HAIC plus TKI and PD-1 might be as follows: 1) HAIC induces tumor antigen exposure through persistent high-concentration chemotherapeutic drug penetration, increases antigenicity through immunogenic cell death of tumor cells, and improves the tumor immune microenvironment to reduce off-targets, thereby enhancing the efficacy of systemic therapy (22); 2) chemotherapy drugs may activate adaptive immunity by increasing leukocyte antigen expression and enhancing T cell stimulation, and restore immune surveillance by disrupting signal transduction and immunosuppression (23); 3) combination of PD-1 inhibitor and anti-VEGF drug may promote normalization of blood vessels breaks the hypoxic microenvironment of tumors and convert cold tumors into hot tumors (24); and 4) anti-angiogenic effects of TKIs and ICIs will help to eliminate tumor angiogenesis and tumor recurrence.

Another strength in this study was that we performed subgroup analysis to verify whether the efficacy of triple therapy would be influenced by other factors. Liver function is the premise of all treatments, and triple therapy means higher requirements for liver function (25). In the present study, we found that Child-pugh A patients had higher DCR (92.2% vs 79.3%, *p*=0.041) than Child-pugh B patients, as well as increased successful conversion rate (22.4% vs 3.4%, *p*=0.019). These findings indicated that liver function must be taken as an important decision-making factor of triple therapy.

Macrovascular invasion and extrahepatic metastasis are both two aggressive hallmarks of HCC, and patients combined with macrovascular invasion or extrahepatic metastasis generally mean adverse prognosis (4). Systematic therapy is the preferred option for patients with macorvascular invasion and/or extrahepatic metastasis, although many novel modalities such as surgical resection combined with local regional therapy have been tried with encouraging results (26, 27). In the present study, subgroup analysis showed that patients with macrovascular invasion have significantly lower rates PR and ORR (both *p*<0.05) compared with the patients with no macrovscular invasion and extrahepatic metastasis, and similar disadvantage was observed in patients with extrahepatic metastasis in terms of ORR and DCR (both *p*<0.05). These results suggested that the current modality might not be appropriate for this population. Considering that tumor thrombus is much more sensitive to radiotherapy (RT), RT-based comprehensive treatment might be ideal option for patients with macrovascular invasion (28). As is known to all, good local control (LC) is positively correlated with improved prognosis, and RT or radiofrequency ablation offer superior LC to ADT for patients with metastasis, such as oligo-metastasis in lung, brain, and bone. As one saying goes, one size does not fit for all. In future, more modalities combined with local treatment and systematic therapy should be worth trying for u-HCC.

As for the choice of the HAIC regimen, there is still no answer. The oxaliplatin-based FOLFOX regimen is currently the mainstream HAIC chemotherapy regimen in China (29), which could regulate the function of immune response, thereby improving the ability of dendritic cells to recognize tumor cells, activating cytotoxic T lymphocytes to attack tumor cells, and leading to tumor immune death (30). While evidences revealed that RALOX was not inferior to FOLFOX in efficacy but with shorter infusion period, which might improve compliance of patients to treatments (31). In the present study, we found that there was no significant differences between the two HAIC regimen in CR, ORR, DCR, and successful conversion rate (all *p*>0.05), which was coincident with previous studies (32). But considering feasibility and compliance, RALOX could be taken as an alternation for selected patients, especially for those with poor performance status.

Safety is a key concern for the triple therapy protocol. In this study, the most common AEs were impaired liver function, myelosuppression, fatigue, nausea and vomiting and abdominal pain. Although the vast majority of patients experienced treatment-related AEs, grade 3/4 treatment-related AEs was 17.7% and no deaths were reported. But this does not mean that we can relax our vigilance. Late onset AEs deserve much more attentions, especially to those related to ICIs. In addition, high rates of impaired liver function damage also indicated the need of adequate liver reserve function for triple therapy, and patients with impaired liver function should be excluded or taken much more carefully.

There were several limitations in this study. Firstly, this was a retrospective, single center, and single-arm study. Although our sample size was large, we did not provide a control group, which is what we need to do next. Second, our treatment regimens were not uniform as depicted in other published studies, which may have some impact on efficacy. Third, median OS could not be derived due to short follow-up time, the choice of subsequent treatment regimens could also have an impact on OS. Fourth, the number of patients in the subgroup analysis may be insufficient, and the conclusions drawn may not be accurate. Prospective randomized controlled trials with large sample sizes are needed to verify the efficacy of triple therapy, and **Table 6** lists ongoing clinical trials.

**Table 6:** Ongoing clinical trials for HCC patients with HAIC+TKIs+PD-1/PD-L1 treatment.

	NCT Number	Phases	Title	Experimental: Treatment Group	Contral Group	Disease stage	Primary end point	Enrollment
1	04961918	Phase 2	The Efficacy of Hepatic Arterial Infusion Chemotherapy (HAIC) Combine Lenvatinib and Durvalumab (HILL) in Advanced Hepatocellular Carcinoma (HCC)	HAIC+Lenvatinib+Durvalumab	None	Advanced HCC	PFS	36
2	05029973	Phase 2	HAIC Combined With Sintilimab and Bevacizumab Biosimilar for Advanced Unresectable HCC	HAIC+Sintilimab+Bevacizumab Biosimilar	None	Advanced unresectable HCC	ORR	30
3	04814043	Phase 2	PD-1 Antibody and Lenvatinib Plus TACE-HAIC for Potential Resectable HCC: a Single-arm, Phase 2 Clinical Trial	Lenvatinib+PD-1 inhibitor	None	Potential resectable HCC	Conversion rate to resection	57
4	05198609	Phase 3	Camrelizumab, Apatinib Plus HAIC Versus Camrelizumab and Apatinib for HCC With Portal Vein Invasion: a Randomized Trial	FOLFOX-HAIC+Camrelizumab+Apatinib	Camrelizumab+Apatinib	HCC With PVTT	OS	214
5	05166239	Phase 2	HAIC Combine With Lenvatinib and Camrelizumab for Advanced HCC With PVTT	HAIC+Lenvatinib+Camrelizumab	Lenvatinib+Camrelizumab	HCC with PVTT	6 months PFS rate	66
6	05135364	Phase 2	HAIC Combined With Camrelizumab and TKI for Unresectable Hepatocellular Carcinoma After TACE Failure	HAIC+TKI+Camrelizumab	None	Unresectable HCC	PFS	48
7	05099848	Phase 2	A Trial of Conversion Treatment of HAIC Combined With Camrelizumab and Apatinib for Unresected Hepatocellular Carcinoma	HAIC+Apatinib+Camrelizumab	None	Unresected HCC	R0 resection rate	20
8	04191889	Phase 2	A Trial of Hepatic Arterial Infusion Combined With Apatinib and Camrelizumab for C-staged Hepatocellular Carcinoma in BCLC Classification	HAIC++Apatinib+Camrelizumab	None	BCLC C-stage HCC	ORR	84
9	04618367	Not Applicable	HAIC Combined With Lenvatinib and Sintilimab for Hepatocellular Carcinoma With PVTT	HAIC+Lenvatinib+Sintilimab	None	HCC With PVTT	PFS rate at 6 months	30
10	05003700	Phase 2	Hepatic Arterial Infusion Combined With Lenvatinib and Camrelizumab for Unresectable Hepatocellular Carcinoma	HAIC+Lenvatinib+Camrelizumab	None	Unresectable HCC	ORR	48

HCC, Hepatocellular Carcinoma; HAIC, hepatic arterial infusion chemotherapy; TKIs, tyrosine kinase inhibitors; PD-1, programmed cell death protein-1; TACE, transarterial chemoembolization; BCLC, Barcelona Clinic Liver Cancer; PVTT, portal vein tumor thrombus; FOLFOX HAIC, Oxaliplatin+Leucovorin+5-fluorouracil; ORR, objective response rate; OS, overall survival; PFS, progression free survival.

## Conclusion

In this real-world series, triple combination of HAIC plus PD-1 inhibitors and TKIs was feasible and efficient in the treatment for patients with initially unresectable HCC. However, more attentions should be paid to screening of potential beneficiary, optimal regimen of triple therapy, timing of treatment response evaluating, standard of successful conversion, subsequent therapy, and late onset AEs. In future, cross-regional centers RCTs with a larger sample size will be helpful in clarifying the role of the triple modality for unresectable HCC.

## Data availability statement

The original contributions presented in the study are included in the article. Further inquiries can be directed to the corresponding authors.

## Ethics statement

The studies involving human participants were reviewed and approved by the Ethics Committee of the first affiliated hospital of Nanchang University, Nanchang, China. No. (2022) CDYFYYLK (06-009). Written informed consent for participation was not required for this study in accordance with the national legislation and the institutional requirements.

## Author contributions

LL, RS and RW: study concept and design. YX, GZ and AH: acquisition and analysis or interpretation of data. LL, TW and XG: drafting of the manuscript. LL, ZH and WW: critical revision of the manuscript. JX and WD: statistical analysis. HM and SS: administrative and technical support. All authors contributed to the article and approved the submitted version.

## Funding

Key R&D Program of Jiangxi Provincial Department of Science and Technology, Grant Number: 20202BBGL73092.

## Conflict of interest

The authors declare that the research was conducted in the absence of any commercial or financial relationships that could be construed as a potential conflict of interest.

## Publisher’s note

All claims expressed in this article are solely those of the authors and do not necessarily represent those of their affiliated organizations, or those of the publisher, the editors and the reviewers. Any product that may be evaluated in this article, or claim that may be made by its manufacturer, is not guaranteed or endorsed by the publisher.
